# Research Progress in Multi-Omics Analysis of Dairy Products: Nutritional Quality, Safety Evaluation, and Health Functions

**DOI:** 10.3390/foods15132389

**Published:** 2026-07-04

**Authors:** Mengqi Xu, Biao Ma, Kaichen Zhu, Wenke Tu, Chenjia Li, Peiying Hao, Mingzhou Zhang

**Affiliations:** Key Laboratory of Microbiological Metrology, Measurement & Bio-Product Quality Security, State Administration for Market Regulation, College of Life Sciences, China Jiliang University, Hangzhou 310018, China; 18036057793@163.com (M.X.); 16a0701109@cjlu.edu.cn (B.M.); zhukaichen163@163.com (K.Z.); 13567598853@139.com (W.T.); lcj1466353561@163.com (C.L.); haopy@cjlu.edu.cn (P.H.)

**Keywords:** dairy products, foodomics, multi-omics integration, nutritional quality, safety assessment, health functions

## Abstract

This review evaluates multi-omics applications in dairy research across nutrition, safety, and health. Through multi-omics integration, we reveal nutrient differences driven by species, rearing practices, and processing techniques, identify protein patterns and allergen profiles, and construct adulteration detection fingerprints and species-specific peptide markers, thereby improving the timeliness and accuracy of safety assessment. The coupling of metagenomics and metabolomics effectively predicts spoilage-related microbial risks, enabling better risk control. Furthermore, multi-omics approaches systematically elucidate the functional mechanisms of bioactive peptides (e.g., ACE-inhibitory peptides), clarify the prebiotic effects of functional oligosaccharides, and build interaction networks between dairy components and gut microbiota. The introduction of machine learning enables origin and shelf-life prediction, as well as the discovery of novel biomarkers, promoting personalized nutrition and precision fermentation strategies. However, the field is currently constrained by severe reproducibility issues arising from the absence of standardized operating procedures, excessive optimism regarding machine learning models that rarely generalize across laboratories or product matrices, and a persistent disconnect between laboratory-scale biomarker discovery and industrial implementation. Without rigorous cross-platform validation and openly shared multi-omics reference datasets, most published markers remain unfit for regulatory or industrial application. Future efforts should establish standardized workflows and expand the evidence base to drive the dairy industry toward safer, healthier, and more traceable directions.

## 1. Introduction

Dairy products are globally important food resources. Their nutritional value encompasses not only basic nutrients, but also a range of bioactive molecules that collectively influence growth, immunity, and metabolic regulation [1]. Although mammalian species are numerous worldwide, commercial dairy products are primarily derived from a limited number of species, such as cattle, goats, and camels [2]. The milk of different species exhibits systematic differences in proteins, lipids, minerals, and other components [3]. These differences provide a molecular basis for the development of customized nutrition and specific processing technologies.

However, the dairy industry faces significant safety and quality challenges. The health risks posed by adulteration practices (such as blending low-cost milk sources with high-value ones) have made efficient and traceable detection systems a regulatory focus [4,5]. Traditional methods such as sensory evaluation, electrophoresis, and immunoassays have limitations in resolution and standardization when dealing with complex adulteration scenarios or deeply processed samples [6].

Against this background, multi-omics technologies represented by proteomics, metabolomics, and metagenomics, combined with bioinformatics analyses (e.g., machine learning, network modeling, molecular docking, etc.), are driving a paradigm shift in dairy research from a “single molecule–single function” to a “multi-molecule network–multi-dimensional function” perspective. These technologies provide systematic tools for non-targeted compositional characterization, source identification, and functional evaluation. For example, LC-MS/MS-based metabolomics, lipidomics, and 4D label-free proteomics have successfully revealed significant differences in metabolites, lipids, and proteins among different dairy products; techniques such as mass spectrometry imaging also play important roles in glycosylation analysis [7,8].

Although multi-omics technologies have achieved significant progress in various scenarios such as dairy species identification, adulteration quantification, thermal damage assessment, fermentation monitoring, and probiotic function prediction, their large-scale application still faces challenges. The main bottlenecks include poor cross-platform data comparability; a lack of international standard operating procedures covering the entire workflow from sampling and sample preparation to analysis and annotation; and an unclear pathway for translating laboratory discoveries into regulatory compliance, process optimization, and health claims.

To overcome the above challenges and fully realize the potential of bioinformatics-driven multi-omics, future research should focus on two major strategic directions. The first is dissecting the molecular mechanism of nutritional quality and safety authentication, i.e., using multi-omics data to identify species-specific protein markers, characteristic lipid clusters, metabolic fingerprints, and functional gene profiles of microbiota, thereby supporting high-resolution traceability and risk early warning. The second is the construction of a systematic validation framework for health functions that combines multi-omics with in vitro cell models, organoids, and clinical cohorts to move from correlation to causal inference.

This review will systematically summarize the latest advances in bioinformatics-driven multi-omics integration for dairy nutritional quality, safety assessment, and health functions in the above directions and propose a research framework for standardization and industrial translation. Multi-omics integration encompasses data-level fusion, methodological integration, and industrial translation.

This review follows the principles of a systematic literature review. Peer-reviewed articles published between January 2015 and March 2026 were retrieved from the PubMed database. The search strategy combined the following keywords: (i) dairy products (such as “milk”, “dairy products”, “yogurt”, “cheese”); (ii) multi-omics technologies (such as “proteomics”, “metabolomics”, “lipidomics”, “metagenomics”, “peptidomics”, “foodomics”); and (iii) application fields (such as “nutritional quality”, “safety”, “adulteration”, “allergens”, “bioactive peptides”, “gut microbiota”). 

Inclusion criteria were as follows: (1) original research or review articles; (2) in Chinese or English; (3) using dairy products as the main matrix; (4) applying at least one omics technology; (5) results involving nutritional quality, safety assessment, or health functions. Exclusion criteria were as follows: (1) non-dairy matrix; (2) unpublished conference abstracts, dissertations, or preprints; (3) only focusing on the detection of a single metabolite, without covering a multi-omics scope. Quality assessment of original research was performed using the observational study standards adapted from the Newcastle–Ottawa Scale and clinical trial standards derived from CONSORT.

## 2. Multi-Omics Analysis for Dissecting the Nutritional Quality of Dairy Products

### 2.1. Species, Environment, and Processing: Three Major Factors Influencing Dairy Product Nutritional Quality

The nutritional quality of dairy products is a complex systemic concept, encompassing multiple dimensions from macronutrients to trace bioactive components. It is mainly influenced by three major factors: species differences, rearing practices (environment), and processing technologies [9]. Milk from different species exhibits inherent differences in proteins, lipids, and minerals, providing a molecular basis for customized nutrition. Rearing practices and environmental stresses can reshape milk composition by modulating rumen microorganisms and host metabolism [10,11]. Thermal treatments, fermentation, and other processing steps further alter protein structures and metabolite profiles, and research to date has failed to comprehensively reveal the holistic impact of the above factors on the molecular composition of dairy products.

**Figure 1:**
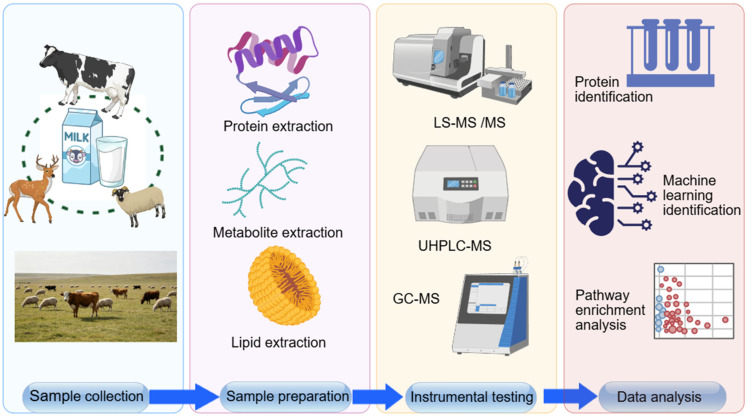
Schematic diagram of the multi-omics detection and analysis process for dairy products. This workflow consists of four sequential stages: sample collection, sample preparation, instrumental testing, and data analysis. Arrows indicate the direction of the analytical pipeline. Different colors distinguish the four stages.

Multi-omics technologies (metabolomics, proteomics, lipidomics, and metagenomics) provide systematic profiling by enabling high-throughput, systems-level profiling (Figure 1) [12,13,14,15,16]. Table 1 summarizes the core targets, application directions, and distinctive advantages of each omics approach.

### 2.2. Nutritional Differences Among Milks from Different Sources: Multi-Omics Fingerprinting with Machine Learning Discrimination

The integrated application of multi-omics technologies, particularly metabolomics, lipidomics, and proteomics, has become a key tool for systematically resolving nutritional differences in dairy products from different milk sources and points to discriminative features amenable to machine learning [15]. By integrating multi-dimensional molecular information from proteomics, metabolomics, and lipidomics, it is possible to construct milk-source-specific molecular fingerprints, providing a high-quality data foundation for subsequent target discrimination based on machine learning.

At the metabolomics level, the application of UHPLC-QTOF-MS, Orbitrap high-resolution mass spectrometry, and 4D label-free quantification technology has enabled deep characterization of metabolite profiles across milk sources from different species [26]. Through systematic analysis of human, porcine, bovine, yak, and buffalo milk, a total of 1992 metabolites were identified, with lipids (21.08%), organic acids and derivatives (18.62%), heterocyclic compounds (14.56%), benzenoids (13.91%), and organooxygen compounds (9.29%) as the major components, providing a high-quality chemical atlas for deciphering the nutritional differences and key biomolecular associations among different milk sources [27]. This study also revealed species-specific molecular networks within individual species, laying a solid data foundation for the subsequent construction of milk-source-specific discriminant models.

At the level of differential comparison, there are systematic differences in protein profiles, metabolite composition, and lipid structures among different milk sources. Rocchetti and O’Callaghan point out that metabolomics technologies have been widely applied in milk discrimination based on factors such as breed, feeding methods, and processing techniques and emphasize that technologies like LC-MS and NMR are driving the evolution from univariate statistical modeling toward multivariate statistics and machine learning modeling, which are more suitable for high-dimensional metabolomics data [28]. At the proteomics level, the expression patterns of casein subtypes and whey proteins exhibit significant species specificity. Their differences not only provide a basis for species identification, but are also closely related to the digestion and absorption characteristics, as well as the functional activity, of the proteins [29]. At the lipidomics level, goat milk is rich in medium-chain fatty acids and polyunsaturated fatty acids, while the lipid profile of cow milk exhibits different distribution characteristics. Similarly, Li et al. employed a multi-omics strategy integrating proteomics, metabolomics, and lipidomics to compare the characteristics of raw milk from Normandy and Holstein cattle [30]. They found systematic differences in proteins, fats, bioactive components, and metabolic pathways and successfully constructed breed-specific molecular fingerprints.

Based on the above multi-omics fingerprint data, the integration of machine learning technologies has fundamentally transformed milk source discrimination from descriptive analysis to predictive modeling. A study reports a novel dairy authentication method based on MALDI-TOF MS lipid–peptide dual-omics fingerprints combined with a machine learning ensemble: the ensemble model constructed from the combined lipid–peptide dataset achieved up to 100% accuracy in organic milk authentication, significantly outperforming models based solely on single-omics feature sets [31]. This result strongly demonstrates that the combination of data fusion strategies and machine learning represents an important trend in multi-omics discrimination systems. Karamoutsios et al. summarize the past decade of studies on species identification and adulteration detection using proteomics and chemometrics, highlighting that multivariate methods such as PCA and PLS-DA are widely used, and that many protein-based biomarkers have achieved detection limits as low as 0.1% or even lower [32].

Collectively, the multidimensional data described above establish milk-source-specific molecular fingerprints, providing a systematic basis for the nutritional quality assessment of dairy products [30,33]. The nutritional differences among dairy products from different milk sources are summarized in Table 2.

### 2.3. Rearing Practices and Environmental Factors: Multi-Omics Joint Analysis Revealing Microbe–Metabolite Associations

The nutritional quality of dairy products is influenced not only by species-related factors, but also by rearing practices and environmental conditions [40]. Multi-omics technologies provide essential tools for elucidating the effects of these exogenous factors on milk composition [41].

Environmental stress, particularly heat stress, can significantly affect the molecular composition of dairy products [42]. Integrated multi-omics studies indicate that heat stress reduces the antioxidant capacity of bovine milk (decreased SOD and GSH-Px activities, increased MDA levels), disrupts energy, amino acid, and lipid metabolism, and leads to a reduction in nutrients such as essential amino acids, unsaturated fatty acids, and polar lipids, while increasing the abundance of spoilage-related microorganisms and significantly elevating off-flavor volatile compounds [43].

Feeding systems also have a significant impact on milk composition. Using metabolomics techniques, studies have found that milk from cows grazing on functionally diverse pastures is enriched with various metabolites associated with enhanced consumer health, whereas milk from cows grazing on single-species pastures does not show this phenomenon, indicating that metabolomics can provide strong evidence for identifying characteristic metabolites of “grass-fed” diets and their health benefits [44]. In terms of feed type, urine metabolomics studies have shown that cows fed with alfalfa (high-quality roughage) exhibit higher milk performance than those fed with corn stover (low-quality roughage). Hippuric acid (HUA) and N-methylglutamic acid (NML-Glu) are key urinary metabolites that distinguish the two feed types, with HUA serving as a biomarker for the metabolic utilization of low-quality roughage [12,45].

At the rumen microbiome level, multi-omics techniques have revealed a close association between the rumen microecology and the nutritional quality of dairy products. Through integrated analysis of metagenomics and metabolomics, researchers found significant differences in the composition and function of the rumen microbiome between cows with high milk protein yield and those with low milk protein yield: the high-yield group was enriched in Prevotella and branched-chain amino acid biosynthesis pathways and exhibited significantly higher concentrations of rumen metabolites (amino acids, carboxylic acids, and volatile fatty acids) [46]. Using linear mixed-effects models, it was estimated that rumen metabolites contributed more to the variation in milk protein yield (29.76%) than microbial composition (17.81%) or microbial function (21.56%) [47].

In terms of functional feed regulation, metabolomics studies have found that supplementation with perilla leaves increases the abundance of pyrimidine nucleotides in rumen fluid and milk and enriches pyrimidine metabolism and unsaturated fatty acid biosynthesis pathways, providing new insights for the development of functional dairy products [48]. Furthermore, feed processing methods also affect milk protein composition: diet type can regulate milk protein synthesis and secretion by influencing rumen microbial protein, yield and the uptake and utilization of amino acids by the mammary gland [49].

In summary, the application of multi-omics technologies has systematically revealed the multi-level mechanisms through which rearing practices and environmental factors influence the nutritional quality of dairy products. Integrated multi-omics strategies provide a systematic tool for gaining a deeper understanding of the associations between these exogenous factors and milk components.

### 2.4. Remodeling of Nutrients by Processing: Multi-Omics Dynamic Network Analysis

During the processing and storage of dairy products, their molecular composition undergoes significant changes. Multi-omics approaches provide powerful tools for systematically dissecting the dynamic changes in nutrients and functional components during processing, as well as for identifying potential markers and reaction pathways [49]. As mentioned earlier, the nutritional quality of dairy products is comprehensively influenced by three major factors—species, rearing environment, and processing technology—and processing technology serves as a key downstream link that determines the quality of the final product.

The use of high-throughput, untargeted multi-omics monitoring strategies during storage has become a cutting-edge approach in this field. A study utilizing a combined ^1^H NMR and UHPLC-QToF/MS platform to analyze metabolite changes in raw milk during freeze-drying and subsequent storage indicated that, during ambient storage, the levels of orotic acid, riboflavin, and acetyl-carbohydrates continuously decreased, while the levels of fatty acids, threonic acid, and uridine showed an upward trend [50]. Subsequent studies further revealed the metabolic profile evolution of spray-dried milk powder under constant temperature and accelerated storage conditions, confirming that storage temperature is a key environmental factor driving this process, thus laying a data foundation for establishing reasonable shelf life and optimizing drying process parameters [50,51].

In the thermal processing stage, multi-omics integration analysis provides molecular-level insights into the effects of different sterilization techniques on milk quality. One study systematically evaluated the differential impacts of industrial-scale direct (130 °C/0.5 s) and indirect (75 °C/15 s) heat treatments on milk protein, metabolite, and lipid profiles, revealing that direct heating is superior to conventional indirect heating in preserving milk proteins and endogenous functional metabolites [52].

In the context of fermented dairy products, the characteristics of lactic acid bacterial strains significantly influence metabolite generation. A study using UPLC-QE-MS untargeted metabolomics to track the fermentation process of a multi-species probiotic culture found that metabolite changes were mainly concentrated in the early stage of fermentation, with various metabolites showing significant correlations with new colony formation, as well as flavor and nutritional quality [53]. Another study compared the contributions of two strains, including Lactococcus lactis LA1 and Lactobacillus helveticus Lh59, to fermentation, revealing strain-specific signature metabolites and interactions, indicating that directed accumulation of effective functional small molecules through the selection of functional lactic acid bacterial strains is a well-founded strategy [54].

During cheese ripening, metabolomics analyses have revealed that tyrosine and its derivatives are closely associated with the degree of ripening, quality, and flavor formation. Biochemical studies indicate that the catabolism of tyrosine (Tyr) and phenylalanine (Phe) by lactic acid bacteria is closely related to the generation of flavor defect compounds such as “barn-utensil” and “floral” notes in cheese [55]. Emerging targeted quantitative multi-omics techniques, such as the LC-MS/MS high-throughput analytical panel covering all 56 glutamyl dipeptides from the α-Glu-X, X-Glu, and γ-Glu-X subgroups, have been successfully applied to precisely assess the effects of cheese ripening, geographic origin, and starter cultures on the contribution to kokumi taste [56].

Furthermore, the initial quality of raw milk is also critical for maintaining processing stability. Targeted lipidomics analysis has revealed that, during cold storage of raw milk at 4 °C for 6 days, lipid molecules such as triglycerides, phospholipids, and free fatty acids undergo metabolic changes that are directly linked to preserving the quality of subsequent processed products [57]. The integrated use of metaproteomics and metabolomics is expected to comprehensively reveal the mechanism of quality evolution during cold storage of raw milk at both the protein and metabolite levels, providing a decision-making basis for developing more effective preservation technologies and optimizing downstream processing techniques [58].

In summary, multi-omics technologies can systematically elucidate the effects of protein degradation, aggregation, and post-translational modifications on the structural properties and nutritional value of dairy products during processing, as well as reveal the dynamic remodeling networks of molecular composition at different processing stages, thereby providing a systematic tool for process optimization and quality enhancement.

### 2.5. Horizontal Comparison: Applicability and Limitations of Different Omics Technologies in Nutritional Quality Research

Each omics technology has its own strengths and weaknesses when applied to assessing the nutritional quality of dairy products. Metabolomics covers small molecules such as organic acids, amino acids, and nucleotides, offering high detection throughput and good sensitivity, making it suitable for large-scale screening. However, it has limited capability for structural identification of unknown metabolites and is susceptible to matrix interference [59]. Proteomics targets caseins and whey proteins, providing high specificity and quantitative accuracy, and is particularly useful for species identification and allergen analysis. Nevertheless, its dynamic range is limited, making the detection of low-abundance proteins challenging. Lipidomics focuses on fatty acids and phospholipids, which are directly related to dairy functionality (e.g., conjugated linoleic acid), but this approach suffers from low standardization, leading to large inter-laboratory variations in lipid identification [23]. Metagenomics reveals microbial composition and functional genes, offering mechanistic insights, but cannot directly reflect the nutritional components of the final product and is relatively costly [60]. Therefore, nutritional quality research should rationally select or integrate multiple omics technologies based on specific objectives: metabolomics for preliminary screening, proteomics for validation, lipidomics for functional targeting, and metagenomics for mechanistic exploration.

## 3. Multi-Omics for Constructing a Dairy Safety Assessment Chain

### 3.1. From Authenticity to Risk Grading: The Progressive Logic of Dairy Safety Assessment

**Figure 2:**
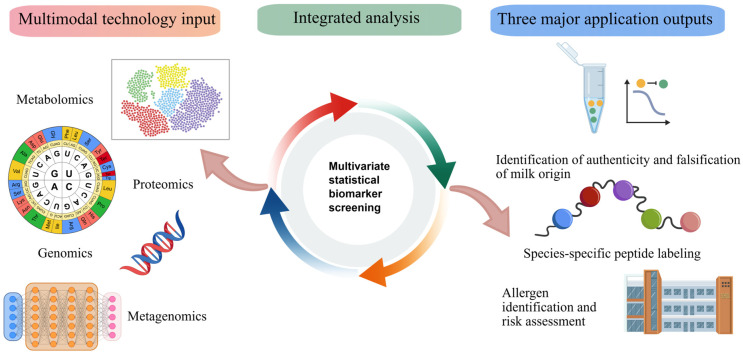
Integrated application framework of multi-omics in dairy product safety assessment. This framework consists of three modules: multimodal technology input (metabolomics, proteomics, and metagenomics), integrated analysis (multivariate statistical biomarker screening, authenticity and falsification identification, species-specific peptide labeling, and allergen risk assessment), and three major application outputs. Arrows indicate the data flow from input through integration to final applications. Color-coded blocks differentiate technology types, analytical methods, and application outcomes.

The previous chapter discussed the application of multi-omics technologies in analyzing the nutritional quality of dairy products. This chapter focuses on recent advances in safety assessment. Multi-omics technologies have shown significant potential in dairy safety assessment, offering insights into quality and traceability (Figure 2) [16]. Their applications mainly cover milk authenticity and adulteration identification, species-specific peptide markers, and allergen recognition and risk assessment [61]. By integrating metabolomics, proteomics, genomics, and metagenomics, a comprehensive evaluation of the nutritional quality, safety authentication, and health functions of dairy products can be achieved. This review summarizes key methods, markers, platforms, models, applicable products, processing conditions, and scenarios for each area, as presented in Table 3.

### 3.2. Adulteration Detection: Multi-Omics Fingerprinting with Database Search for Species-Specific Peptides

As globally consumed nutritional commodities with high economic value, dairy products are frequently subjected to adulteration, especially by mixing with lower-cost milk types [32]. Multi-omics technologies integrate multidimensional molecular information from the proteome, peptidome, metabolome, genome, and others to construct milk-source-specific molecular fingerprinting profiles. Combined with database searching and machine learning algorithms, this approach enables high-sensitivity detection of adulterants and species origin tracing [72].

Mass spectrometry-based proteomics and peptidomics are among the core technologies for adulteration detection [73]. Whey proteins (such as β-lactoglobulin and α-lactalbumin) and casein subtypes exhibit systematic differences across species. Owing to their high thermal stability and processing tolerance, peptides offer greater value than intact proteins for species identification in highly processed dairy products [74]. Using quantitative proteomics and metabolomics, Ji et al. found that protein biomarkers (e.g., OPN, TF) detected bovine milk adulteration at 1% in pasteurized mare milk but only 10% in mare milk powder, whereas N6-methyladenosine detected 0.1% in powder. They also noted that processing reduces biomarker sensitivity, necessitating method adaptation for different product forms [63]. Another study utilized proteomics combined with artificial neural network technology to analyze thousands of mass spectra of cow, goat, and sheep milk collected by MALDI-TOF MS. Through machine learning algorithms, it effectively distinguished the species-specific mass spectral features, providing a high-precision solution for dairy product authentication [75].

In terms of integrating peptidomics and proteomics, a MALDI-TOF-MS-based integrated platform enables simultaneous peptidomic and proteomic analysis. By identifying protein or peptide markers specific to buffalo, goat, or sheep milk, it can rapidly detect whether undeclared cow milk has been adulterated and estimate the extent of adulteration [76,77]. This method has also been extended to characterize heat treatment markers. Compared with earlier approaches that relied solely on protein mass spectrometry, complementary peptide profiling measurements further validate the results and broaden the applicability [78].

Metabolomics also provides important clues for adulteration identification [28]. The species-specific profiles of endogenous small molecules (such as nucleotides, organic acids, and lipids) can be captured by NMR, GC-MS, and LC-MS platforms. Combined with multivariate statistical methods such as partial least squares discriminant analysis, they can effectively distinguish milk subjected to different intensities of heat treatment, thereby aiding in the identification of adulteration [26]. A study systematically reviewed the effects of intrinsic factors (cattle breed, lactation stage) and extrinsic factors (feed, season, region, processing, and storage) on milk metabolites, noting that these metabolite changes can serve as potential biomarkers for evaluating milk origin traceability and quality [26,79].

At the genomics level, by targeting mitochondrial DNA or single-nucleotide polymorphism sites in nuclear genes (such as the casein gene CSN1S1 and the whey protein gene LALBA), precise species tracing and genetic background verification of dairy products can be achieved [80]. Metagenomics can detect residual host cell DNA and symbiotic microbial community structure in raw milk, assisting in ruling out illegal mixing or dilution [81].

Based on the aforementioned multi-omics fingerprints, the integration of machine learning algorithms shifts adulteration identification from descriptive analysis to predictive modeling [82]. Chemometric methods, such as principal component analysis and partial least squares discriminant analysis, are increasingly being integrated into proteomics workflows to handle high-dimensional datasets [32]. Recent studies have explored the application of various machine learning techniques in dairy adulteration detection. For example, a multi-functional detection method using hyperspectral imaging combined with models such as logistic regression, decision trees, and support vector machines achieved high discrimination accuracy on validation sets [83]. Gradient boosting algorithms, represented by XGBoost and LightGBM, have demonstrated classification performance in adulteration detection [84]. However, the lack of standardized protocols, high variability in sample preparation, and insufficient cross-breed and cross-region validation remain core challenges currently faced.

In terms of machine learning algorithm selection, different models exhibit significant differences. Support vector machine (SVM) is robust with small sample sizes but has limited capacity for fitting nonlinear relationships [85]. Random forest (RF) can handle high-dimensional features but is prone to overfitting, especially when the number of features far exceeds the number of samples [86]. XGBoost and LightGBM achieve the highest AUC in most datasets, yet they are sensitive to hyperparameters and require relatively large training sets [87]. Deep learning models (e.g., CNN) demonstrate feature extraction capabilities for spectral and peptidomic data; however, their “black-box” nature, dependence on massive training samples, and poor cross-platform transferability limit their application in regulatory scenarios. Currently, there is no universally optimal algorithm. It is recommended to adopt ensemble strategies or multi-model voting mechanisms based on sample size, feature dimensionality, and detection context. Furthermore, explainable artificial intelligence (e.g., SHAP values) is emerging as an effective tool for screening key biomarkers and reducing model complexity [88].

In summary, multi-omics fingerprints (metabolomics, proteomics, peptidomics, genomics, metagenomics) combined with database searching and machine learning discrimination provide an integrated analytical pipeline from molecular characterization to machine learning classification model for dairy product adulteration detection. In the future, it is necessary to further establish standardized workflows and multi-center validation systems to promote the practical application of this technology in production enterprises and regulatory authorities [75,78].

### 3.3. Allergen Identification: Multi-Omics Combined with Immunoinformatics for Sequence Alignment and Epitope Prediction

Allergen identification is key to the accurate diagnosis of and effective intervention for allergic diseases [89]. Dairy allergy, particularly cow’s milk allergy (CMA), is one of the most common food allergies in infants worldwide. The major allergens include the casein family (αS1-, αS2-, β-, and κ-casein) and whey proteins (β-lactoglobulin, α-lactalbumin) [90]. In recent years, multi-omics technologies, by integrating data from different levels such as proteomics, metabolomics, and metagenomics, have provided systematic tools for the comprehensive characterization of allergens in dairy products and the assessment of their allergenicity [91,92].

Mass spectrometry-based proteomics has become a core technology for the accurate detection and quantification of allergens. Xu et al. employed bottom-up proteomics combined with high-resolution mass spectrometry to develop a targeted quantitative method for the simultaneous detection of six cow’s milk allergens (α-lactalbumin, β-lactoglobulin, αS1-casein, αS2-casein, β-casein, and κ-casein) in hypoallergenic formulas. They screened and validated 15 signature peptides and ultimately selected 6 peptides for quantitative analysis [93]. This method outperformed both literature-reported and VITAL thresholds and successfully detected allergen residues in commercially available partially and extensively hydrolyzed formulas, providing a direct tool for the quality control of hypoallergenic formula products [92,93].

At the level of epitope recognition and allergenicity assessment, the application of multi-omics approaches has achieved significant advances. Shen et al. developed a highly sensitive, wide-range quantitative method based on IgE multi-epitope-specific antibodies for detecting bovine αS1-casein in foods and predicting potential milk allergenicity [94]. Using polyclonal antibodies against nine IgE epitopes of αS1-casein as detection probes, they established both a conventional sandwich enzyme-linked immunosorbent assay (ELISA) and an Fe-N-C single-atom nanozyme probe sandwich ELISA. The method demonstrated good performance in terms of sensitivity, specificity, accuracy, precision, and recovery, with a linear detection range spanning four to five orders of magnitude [94]. A study involving 118 Spanish children with CMA used a peptide microarray to detect specific IgE/IgG4 linear epitope binding of five major cow’s milk allergens. Combined with baseline serological variables, an XGBoost algorithm was employed to establish a tolerance prediction model [95]. The model achieved AUCs of 0.883 and 0.833 at 6 and 30 months, respectively, indicating that baseline epitope binding profiles can effectively predict when affected children will acquire tolerance [95].

At the level of multi-omics-integrated risk assessment, the integration of multi-omics data with machine learning models has opened new avenues for personalized allergy risk assessment. Hendrickx et al. employed a late-integration multi-view learning approach to fuse clinical data, microbiome data, metaproteomic data, immune data, and metabolomic data, constructing a machine learning classifier capable of predicting CMA remission in infants [91]. The results showed that integrating multi-omics data significantly improved classification performance for CMA remission compared to using single data types alone. Notably, the integration of microbiome data with clinical, immune, metaproteomic, and metabolomic data substantially enhanced classification accuracy in infants with CMA [91]. Furthermore, pathways previously associated with CMA development were also linked to allergy remission, providing new insights into intervening in the allergic process through modulation of host–microbe interactions.

In summary, mass spectrometry-based proteomics provides reliable technical means for high-throughput, high-sensitivity quantitative detection of allergens; the integration of metabolomics and metagenomics offers new molecular-level insights into understanding allergic mechanisms and identifying biomarkers; and risk assessment models integrating multi-omics with machine learning are driving allergy research from association analysis toward clinical applications and personalized prediction.

### 3.4. Risk Assessment: Integration of Multi-Omics Markers and Microbial Early-Warning Models

Dairy product spoilage and pathogenic microorganism contamination pose serious challenges to global food safety [96]. Traditional risk assessment methods rely on conventional microbiological approaches based on shelf-life studies or evaluations of specific spoilage organisms [97]. However, these data are typically generated using traditional methods and often overlook critical information such as antimicrobial resistance, biofilm formation, and virulence factors, as well as the complex interactions among the biochemical characteristics of the food matrix [98]. Moreover, microbial risks in food are diverse and heterogeneous, potentially resulting from the growth and activity of multiple microbial populations rather than contamination by a single species, which limits the efficiency of traditional risk assessment [99]. Against this background, the integration of multi-omics technologies provides revolutionary systemic tools for risk assessment in the dairy industry [100]. By employing metagenomics to identify the composition and function of spoilage and pathogenic microorganisms, metabolomics to synchronously monitor the accumulation kinetics of spoilage markers, and proteomics to elucidate microbe-driven protein degradation mechanisms, multi-omics integrated strategies are shifting from traditional qualitative or quantitative risk assessment toward molecular evidence-based precision risk assessment [61].

In addressing the issue of microbe-driven deterioration of the quality of raw milk during cold storage, multi-omics technologies have demonstrated great potential for in-depth analysis of the community structure and function of spoilage microorganisms [101]. A study using metagenomics and proteomics to analyze raw milk samples stored at 4 °C for 6 days revealed that the relative abundance of Pseudomonas increased significantly with extended cold storage time, and, together with Acinetobacter, it became the dominant bacterial genus in milk after 6 days [102]. The analysis showed that differential proteins in the early stage of cold storage were mainly involved in immune regulation and signal transduction, while those in the later stage were primarily associated with carbohydrate and lipid metabolism [102]. Another study using coupled metagenomic and metabolomic analysis found that, during storage of raw milk at 4 °C for 3–4 days, the microbial community shifts from Acinetobacter and other species toward Pseudomonas, with the most significant metabolic changes coinciding with the rapid growth phase of psychrophilic bacteria, representing a critical window for quality control [103].

Regarding the dynamic interactions between microbiota and metabolites during the storage and processing of raw milk and dairy products, a study using metagenomic next-generation sequencing combined with LC-MS and GC-MS analyzed the changes in microbiota and metabolites during storage of raw milk, pasteurized milk, and UHT milk. The results showed that the succession trends differed significantly between raw milk and heat-treated dairy products. In refrigerated pasteurized milk, Microbacterium, unclassified Actinomycetia, and Micrococcus showed the most significant increases in abundance and were highly correlated with multiple metabolites, indicating that these genera are the predominant proliferating and metabolically active microbiota [104]. This integrated multi-omics analysis strategy provides a new perspective for systematically studying interacting factors during dairy processing and storage. A study using metagenomics and proteomics validated that the microbe–metabolite–protein association network can be used to construct a risk assessment model for quality deterioration of refrigerated milk. Metagenomics revealed an increase in Pseudomonas abundance, while proteomics showed that proteins in the later stage of cold storage were enriched in carbohydrate and amino acid metabolic pathways [102].

In summary, the deep integration of multi-omics with metagenomics, metabolomics, and proteomics is driving the transformation of dairy risk assessment from traditional, empirical-based linear models toward a precision assessment framework grounded in molecular evidence. By integrating multi-level data from metagenomics, proteomics, and metabolomics to construct microbe–metabolite–protein association networks, spoilage-related microbial and metabolic biomarkers can be identified, providing early warning signals for dairy spoilage risk. However, matrix effects from complex food matrices, insufficient cross-region and cross-breed validation, lack of data standardization, and detection stability in industrial application scenarios remain core challenges that must be overcome for practical implementation in this field [15,96].

## 4. Multi-Omics for Revealing the Mechanisms of Dairy Health Functions

### 4.1. From Molecules to Gut Microbiota: Hierarchical Dissection of Dairy Health Functions

From a foodomics perspective, research on the health functions of dairy products is shifting from the identification of single bioactive components toward multi-level, multi-scale systematic analysis. Components such as bioactive peptides, functional oligosaccharides, and lipids found in dairy products can exert various health benefits—including antioxidant, antihypertensive, immunomodulatory, and intestinal barrier repair effects—by directly acting on host targets or indirectly modulating the gut microbiota and its metabolites [105].

This chapter will elaborate on the latest advances in multi-omics applied to the study of dairy health functions from three progressive levels. First, at the molecular level, the integration of peptidomics with molecular docking simulations enables efficient mining of bioactive peptides with angiotensin-converting enzyme (ACE)-inhibitory, antioxidant, and other activities, as well as prediction of their binding modes to target proteins. Second, at the metabolic pathway level, the combined analysis of metagenomics and metabolomics can elucidate the prebiotic effects of functional oligosaccharides, revealing how they modulate gut microbial metabolism to produce beneficial metabolites such as short-chain fatty acids (SCFAs). Finally, at the ecosystem level, multi-omics co-occurrence networks and causal inference models can construct complex interaction maps between dairy components and the gut microbiota, identifying key microbial taxa and metabolic signaling pathways that drive host health. This hierarchical strategy provides a scientific basis for developing precision nutrition strategies and functional dairy products [106].

To provide a concise overview of the multi-omics approaches discussed in the following Section 4.2, Section 4.3 and Section 4.4, Table 4 summarizes the multi-omics technologies and strategies applied at the molecular, metabolic pathway, and ecosystem levels to elucidate the health functions of dairy products.

### 4.2. Bioactive Peptide Mining: Multi-Omics Integrated Molecular Docking and Function Prediction

Milk-derived bioactive peptides are natural functional fragments embedded within the amino acid sequences of milk proteins, typically composed of 2 to 20 amino acid residues [107]. Released through gastrointestinal digestion, in vitro enzymatic hydrolysis, or microbial fermentation, they can exert various physiological functions such as antioxidant, antihypertensive, antimicrobial, and immunomodulatory effects [108]. Traditional approaches rely on an orthogonal strategy involving in vitro enzymatic hydrolysis, chromatographic separation and purification, and activity screening, which suffer from inherent limitations including long turnaround times, low throughput, and high costs [109,110]. The integrated strategy combining omics and computational simulation is profoundly reshaping this paradigm, upgrading the discovery of active components—such as antihypertensive (ACE inhibitory) peptides, hypoglycemic (DPP-IV inhibitory) peptides, and antioxidant peptides—from empirical experimental screening to data-driven rational design [111].

In the realm of databases and computational prediction, the BIOPEP-UWM database is the most widely used bioactive peptide information platform in food science [112]. It comprises four main modules––a protein sequence database, a bioactive peptide sequence database, an allergenic peptide and epitope database, and a sensory peptide database––thereby laying the foundation for systematic knowledge of milk-derived active peptides [113]. A study using this database tool predicted that bovine milk proteins, after treatment with the three enzymes, could generate 59 bioactive peptides with ACE-inhibitory, DPP-IV-inhibitory, and antioxidant activities [114]. Further validation using the INFOGEST in vitro simulated digestion model identified 36 of these peptides across three milk protein fractions, confirming good correspondence between the computational predictions and the in vitro digestion results [115]. Meanwhile, machine learning-based functional prediction models are also being gradually integrated into this workflow. One study trained five machine learning models—GBDT, XGBoost, LightGBM, CatBoost, and random forest—to predict DPP-IV-inhibitory activity, with the LightGBM model achieving the best performance [116]. This model was then applied to large-scale prediction of DPP-IV inhibitory peptides generated from in silico enzymatic hydrolysis of bovine milk proteins, successfully identifying two candidate peptides, GPVRGPF and HPHPHL, with promising activity [116].

In the experimental identification and characterization of peptide sequences, liquid chromatography–tandem mass spectrometry (LC-MS/MS) platforms based on high-resolution mass spectrometry have become the mainstream tool for identifying the sequences of milk-derived bioactive peptides [117]. Labeled quantitative peptidomics analysis of human milk, bovine colostrum, and donkey colostrum compared the differences in major proteins and their released endogenous peptide profiles, providing a basis for understanding the composition patterns of bioactive peptides in colostrum from different species. On this basis, a study integrated simulated gastrointestinal digestion, proteomics, and computational prediction tools to identify three novel peptides with distinct biological activities from caseins. Among them, EMPFPKY (βCN) exhibited antioxidant activity in vitro and was predicted by molecular docking to bind to the Keap1 protein; FVAPFPEVFG (αCN) showed dual ACE-inhibitory and anticancer activities against MCF-7 breast cancer cells; and NLLRF (αCN) demonstrated selective toxicity toward cancer cells while preserving normal cell activity. These findings illustrate the synergistic advantage of combining experimental and computational approaches to identify and characterize multifunctional peptides [118].

Molecular docking and mechanism validation are central to verifying biological activity and elucidating molecular mechanisms. In a study on the synthesis and in vitro activity characterization of ACE-inhibitory tripeptides and pentapeptides derived from bovine milk proteins, molecular docking analysis not only revealed the binding modes of these peptides to the S1 and S2 pocket regions and the zinc ion domain of ACE, but also elucidated the mechanism by which they block ACE catalytic function through competitive inhibition. Hydrogen bonds were identified as the main driving force maintaining molecular interactions, and all three peptides maintained relatively stable ACE inhibitory activity after simulated gastrointestinal digestion [119]. In a study on the ACE inhibitory activity and molecular docking of heptapeptides derived from bovine κ-casein hydrolyzed by chymosin, trypsin, and pepsin, molecular docking simulations further indicated that oxyanion and cationic hydrogen bonds play important roles in the stable binding of the heptapeptides to the ACE active center, and the proline residue at the C-terminus is crucial for maintaining the inhibitory activity of the peptides [120]. The docking and simulation results of YKVPQLEIVP derived from bovine milk β-casein and its analogs further indicate that the contributions of key amino acid residues Leu, Val, and Pro to DPP-IV inhibitory activity cannot be neglected, providing theoretical guidance at the structural level for subsequent rational design and structural optimization [121].

In terms of peptide function screening approaches, the three mainstream strategies each present their own strengths and limitations. Molecular docking provides structural information on the binding modes between peptides and target proteins (e.g., ACE, DPP-IV), identifying key residues involved in hydrogen bonds, hydrophobic interactions, etc. [122]. However, it relies on known crystal structures and cannot predict novel targets or dynamic conformational changes [123]. Machine learning-based prediction (e.g., LightGBM, random forest) enables high-throughput screening of thousands of peptides with low cost and high speed, but the predicted results require in vitro validation, and the generalizability of models varies considerably depending on the training dataset [124]. In vitro experiments (including enzyme activity inhibition assays and cell models) represent the gold standard for functional validation, yet they suffer from low throughput, long time consumption, and high cost [125]. The integrated strategy combining these three approaches—machine learning-based preliminary screening to narrow down candidates, followed by molecular docking to decipher the binding mechanism, and finally in vitro experiments to validate activity—is currently recognized as the optimal pathway [126]. This strategy has been successfully applied to the efficient discovery of ACE-inhibitory peptides and DPP-IV-inhibitory peptides.

In summary, the integrated multi-omics computational-assisted mining paradigm—using machine learning models and peptidomics as high-throughput prediction and experimental identification tools, and molecular docking as a mechanism validation approach—not only accelerates the identification efficiency of bioactive peptides with potential functions such as ACE-inhibitory, DPP-IV-inhibitory, and antioxidant activities, but also provides reliable methodological support for systematic structure–activity relationship analysis and the precision development of functional dairy products.

### 4.3. Prebiotic Effects of Functional Oligosaccharides: Multi-Omics Combined Metabolic Pathway Enrichment Analysis

**Figure 3:**
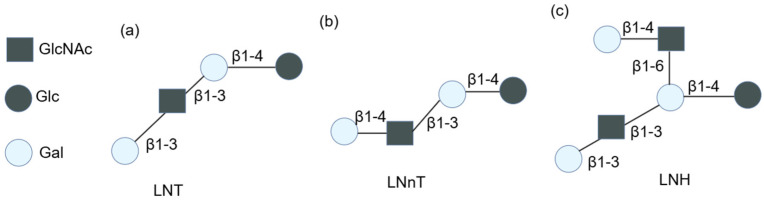
HMO structure diagram (**a**–**c**).

**Figure 4:**
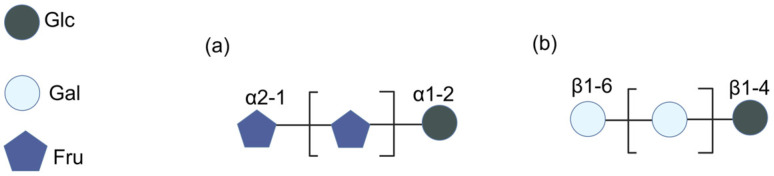
Schematic diagrams of FOS and GOS structures. (**a**) Schematic diagram of FOS structure. (**b**) Schematic diagram of GOS structure.

Functional oligosaccharides are a class of carbohydrates that are not digested or absorbed by the host’s gastrointestinal tract but can be selectively utilized by gut microbiota and confer health benefits to the host [127]. They include human milk oligosaccharides (HMOs), fructo-oligosaccharides (FOSs), and galacto-oligosaccharides (GOSs), among others (see Figure 3 for HMO structure, and Figure 4a and Figure 4b for FOS and GOS structures, respectively). In recent years, multi-omics technologies, particularly the combined application of metagenomics and metabolomics, have become core research tools for systematically elucidating the prebiotic effects of functional oligosaccharides and their patterns of enrichment in metabolic pathways [128].

As the third most abundant solid component in breast milk after lactose and fat, HMOs play an indispensable regulatory role in the assembly of the infant gut microbiota and the shaping of metabolic functions. A study integrating metagenomics and metabolomics found that human milk oligosaccharides (2′-FL, LNT, 3′-SL) can modulate the infant gut microbiota (increasing Bifidobacterium, Lactobacillus, and Enterococcus, while reducing Escherichia–Shigella), significantly perturb amino acid, purine, and lipid metabolic pathways, and elevate short-chain fatty acid levels, revealing that HMOs are associated with prebiotic effects, as indicated by enrichment of microbiota-linked metabolic pathways [129].

Fructo-oligosaccharides (FOSs) selectively promote beneficial gut microbes, enhance immune responses, and increase SCFA production. A study integrating metagenomics and metabolomics in an FOS supplementation trial found substantial inter-individual variability in gut microbial composition and function: the increase in Bifidobacterium abundance was significantly greater in some participants than in others, which was associated with differences in the expression of genes encoding enzymes involved in fructose metabolic pathways; metabolomics further revealed individualized metabolic responses in fructose utilization patterns [130]. Pulse metatranscriptomics, by tracking the transcriptional response dynamics of gut microbiota to fructo-oligosaccharides, elucidated an induced expression cascade of CAZymes and identified multiple atypical FOS-metabolizing strains, providing key support for deciphering their metabolic profiling and prebiotic effects [131].

In terms of functional elucidation of galacto-oligosaccharides (GOSs), a double-blind randomized controlled trial showed that, after 4 weeks of intervention with GOS-enriched low-lactose milk, responders had reduced symptom scores, while non-responders showed increased Bifidobacterium abundance; plasma metabolomics identified 12 differential metabolites (mainly involved in amino acid and lipid metabolic pathways), indicating that individualized baseline gut microbiota composition determines the response to GOS intervention, providing feasibility evidence for precision prebiotic intervention [132].

In summary, multi-omics studies on human milk oligosaccharides, fructo-oligosaccharides, and galacto-oligosaccharides collectively demonstrate that the combined analysis of metagenomics and metabolomics can systematically reveal the regulatory effects of oligosaccharides on gut microbiota composition, short-chain fatty acid metabolism, and amino acid and lipid pathways, thereby providing a technical framework for elucidating the molecular mechanisms of prebiotic effects and for precision nutrition intervention.

### 4.4. Dairy–Gut Microbiota Interactions: Multi-Omics Co-Occurrence Networks and Causal Inference

The interaction between dairy components and the gut microbiota is not a simple linear relationship, but rather involves multi-level synergistic changes within the microbiota, between the microbiota and metabolites, and between metabolites and host phenotypes [133]. Traditional correlation analysis can only capture pairwise statistical dependencies and is insufficient to reveal the network structure of multi-party interactions in complex ecosystems, often misinterpreting indirect associations as direct causality [134]. The integration of multi-omics co-occurrence networks and causal inference methods provides a systematic tool to address this challenge [135].

Co-occurrence network analysis, by constructing an association map among microbes, metabolites, and host indicators, can identify key functional modules driving the health effects of dairy products. A randomized controlled study in healthy adults showed that, after an 8-week intervention with probiotic fermented milk, the density of the gut microbiota–metabolite co-occurrence network and the number of connections related to anti-inflammatory metabolites increased, and the Lactobacillus–Bifidobacterium–SCFA module was positively correlated with IL-10. This indicates that fermented milk enhances the functional stability of the microbiota by remodeling the network structure [136]. In a study of gut microbiota enterotypes in dairy goats, co-occurrence network analysis also revealed complex interactions between different microbial community patterns and lactation performance, as well as differences in key species [136]. A systematic review further integrated multi-omics evidence, indicating that fermented dairy products synergistically enhance microbial diversity, strengthen the intestinal epithelial barrier, and modulate immune signaling pathways through live cultures and their metabolites. However, current research is limited by heterogeneous experimental designs and short intervention periods, highlighting the urgent need to integrate longitudinal multi-omics data to establish persistence and causal validation [128].

In terms of causal inference, Mendelian randomization uses genetic variants as instrumental variables to infer the causal direction between dairy consumption and gut microbiota in observational studies. A bidirectional Mendelian randomization study in a European population, using the LCT gene variant as an instrumental variable, found that a genetically predicted increase in milk intake led to increased abundances of Bifidobacterium and Lactobacillus and decreased abundances of Streptococcus and Escherichia/Shigella, with no significant reverse causality, supporting a unidirectional causal relationship of “dairy intake driving microbiota remodeling.” This study further extended the analysis of the association between LCT variants and gut microbiota as a function of dairy intake [137]. Furthermore, a study using two-sample Mendelian randomization analyzed the causal relationship between milk intake and body mass index, as well as blood lipids, providing genetic evidence for causal inference regarding the impact of dairy consumption on metabolic phenotypes [138].

Structural equation modeling can simultaneously evaluate direct and indirect pathways among multiple latent variables and is suitable for dissecting the multi-level causal chain of “dairy products–gut microbiota–host health.” A review by Kinkpe et al. systematically summarized the application of network analysis combined with multi-omics data in identifying key species, emphasizing that multi-omics integration is a key approach to separating true ecological relationships from statistical noise and linking microbes to functions, thereby providing methodological support for constructing a microbiota–metabolite–host health network framework [139]. Martinez-Boggio et al. utilized structural equation modeling and mediation analysis to investigate the effects of the host genome and rumen microbiome on feed efficiency in dairy cows, demonstrating a practical application of mediation-based structural equation modeling in inferring microbe-mediated hierarchical causal pathways [138]. Li et al. employed a multi-omics approach combined with mediation analysis and causal network inference to systematically dissect the microbial metabolic pathways and causal links through which probiotic fermented milk remodels the gut microbiota and exerts angiotensin-converting enzyme (ACE) inhibitory functions, demonstrating the feasibility of the multi-omics causal analysis framework in moving from correlation to mechanistic validation [140].

Most studies cited in this section report correlational associations rather than definitive causal mechanisms. Mendelian randomization and structural equation modeling can suggest directionality, but they rely on strong assumptions (e.g., no horizontal pleiotropy) that are rarely fully met in dairy nutrition studies. Gold-standard causal evidence would require randomized controlled trials with multi-omics endpoints, which remain scarce. In summary, co-occurrence network analysis reveals functional association patterns between dairy components and the gut microbiota, while causal inference methods such as Mendelian randomization and structural equation modeling translate these associations into testable causal pathways. The multi-omics integrated causal analysis framework is advancing research on dairy–gut microbiota interactions from correlational description toward mechanistic validation, providing actionable scientific evidence for precision nutrition interventions based on the gut ecosystem and for the development of functional dairy products.

## 5. Data Integration and Standardization Challenges: Toward Reproducible Dairy Omics Research

### 5.1. Cross-Platform Data Standardization and Multi-Omics Workflow Construction

Moving from laboratory discoveries to industrial applications faces multiple challenges [140]. One of the most prominent bottlenecks is the lack of standardization in multi-omics data preprocessing. Aside from the absence of unified standard operating procedures, persistent unresolved technical obstacles including unpredictable batch effects stemming from long-term instrument drift without adequate pooled reference samples or randomized sequencing arrangements, inconsistent sample preparation workflows across labs that compromise result repeatability, divergent outcomes from varied peak-processing algorithms and insufficient documentation of relevant software settings, uneven metabolite database annotation stemming from disparate spectral repositories and insufficient verification against authentic reference compounds, and poor cross-platform compatibility among LC-MS, NMR, and GC-MS datasets paired with benchmark-deficient integration approaches reliant on subjective parameter adjustment for dairy-related samples collectively demand joint efforts from the research community to develop standardized reference materials, enforce compliance with established reporting guidelines such as MIAPE and metabolomics standards initiative checklists, and launch dedicated interlaboratory ring tests targeting dairy matrices to achieve effective resolution [141].

Closely related to this is the specialized demand for bioinformatics expertise: multi-omics datasets are typically large in scale and highly heterogeneous, and their storage, processing, and analysis require interdisciplinary knowledge of topics including biostatistics, machine learning, programming, and biology [15]. Developing customized bioinformatics pipelines that incorporate different methods, flexible parameter settings, and robust version control remains a major bottleneck in this field [61].

Furthermore, the selection of integration methods is also considerably complex. Currently, a variety of multi-omics integration methods exist (such as MOFA, DIABLO, SNF, etc.), each employing different algorithmic strategies [142]. The lack of a general framework to guide which method is most suitable for a given dataset or biological question often leaves researchers confused [143]. Even when data integration is successfully completed, translating algorithmic outputs into actionable biological insights remains a significant bottleneck. In dairy research, this specifically manifests as difficulty in directly linking multi-omics findings to milk quality indicators, health functions, or production process optimization [144].

To overcome the above challenges, a standardized framework for dairy multi-omics research needs to be established. At the data level, a cross-platform molecular reference database (e.g., LactoBase) should be built to collect species-specific protein markers, characteristic lipid clusters, metabolic fingerprints, and functional gene profiles of microbiota, while promoting unified formats for public data repositories to ensure that data are Findable, Accessible, Interoperable, and Reusable (FAIR principles) [145]. At the methodological level, joint efforts by ISO and the International Dairy Federation (IDF) should be promoted to develop multi-omics standard operating procedures (SOPs) covering the entire workflow from sample collection, pretreatment, and mass spectrometry analysis to data annotation, with differentiated pretreatment and analysis parameters tailored to different dairy matrices (liquid milk, milk powder, fermented milk) [146]. At the application level, a closed-loop translation pathway from omics discovery to biomarker validation, regulatory approval, and industrial adaptation needs to be established, simplifying complex omics workflows into low-cost, easy-to-operate rapid detection solutions that can be adopted by small and medium-sized dairy enterprises [147].

Despite the proliferation of high-accuracy claims, the reality is that the vast majority of published multi-omics biomarkers fail in independent validation. This is rarely disclosed in the literature. The reasons are structural: (i) batch effects across mass spectrometry platforms are consistently underestimated, with day-to-day instrument drift alone often exceeding biological variation [148]; (ii) sample sizes in most dairy omics studies are grossly insufficient for the dimensionality of features (thousands of metabolites/peptides), leading to massively inflated classification performance through overfitting [149]; (iii) machine learning models are frequently trained and tested on data from the same batch or same farm, yet their performance collapses when applied to milk from different seasons, regions, or processing conditions [148]; (iv) publication bias strongly favors positive results, while negative validation studies remain largely unpublished or buried in supplementary materials [150].

Consequently, claims such as “achieved 100% accuracy for organic milk authentication” or “LOD as low as 0.1% for adulteration” must be read with caution: they are typically derived from convenience samples under optimal laboratory conditions, not from the heterogeneous, uncontrolled environment of routine industrial quality control. The field urgently needs mandatory cross-laboratory ring trials and pre-registered validation protocols before any multi-omics signature can be considered for standardization [151].

### 5.2. Multi-Omics Machine Learning Prediction Models: From Biomarkers to Personalized Nutrition and Precision Fermentation

Integrating machine learning with multi-omics is one of the key pathways to overcome the above bottlenecks. Traditional correlation analysis primarily captures linear relationships and struggles to represent the complex nonlinear interactions in biological systems. Machine learning algorithms (e.g., random forest, support vector machines, and XGBoost) can handle high-dimensional data and identify more robust and biologically relevant combinations of biomarkers [152]. Deep learning models (e.g., deep autoencoders, hypergraph convolutional networks) can automatically learn complex features and patterns in data, making them particularly suitable for processing high-dimensional, nonlinear multi-omics data [153]. In dairy research, deep learning can be used to predict molecular changes in products under different processing conditions, identify key biomarker combinations affecting quality and safety, and build end-to-end prediction models from multi-omics data to final product attributes [142].

However, the practical reality of machine learning in dairy multi-omics is far more sobering. Published models rarely report performance on truly independent external test sets (e.g., from different countries or different processing plants) [154]. When such tests are performed, the area under the curve (AUC) typically drops by 0.2–0.4 compared to internal cross-validation [155]. Moreover, most models are not reproducible: the combination of feature selection, hyperparameter tuning, and random seed variance leads to entirely different sets of “key biomarkers” when the same analysis pipeline is re-run on the same data [156]. This “instability of discovery” directly undermines the credibility of multi-omics for regulatory use. A few critical limitations that are systematically underreported include (i) the fact that the sample-to-feature ratio rarely exceeds 1:100, making deep learning models almost certain to memorize noise; (ii) the lack of prospective validation—almost no dairy omics model has been tested in a real production line with continuous on-line monitoring; (iii) the black-box nature of the analysis– even when SHAP values are provided, they do not guarantee biological causality, and regulatory agencies remain reluctant to accept non-mechanistic models for safety decisions. Future work must prioritize small, interpretable feature sets validated across at least three independent laboratories and on at least two different mass spectrometry platforms before any industrial deployment is considered.

Based on the above analysis, future research should focus on two major strategic directions. The first direction is the molecular mechanism dissection of nutritional quality and safety authentication, namely, using machine learning to build a machine learning classification model for species-specific protein markers, characteristic lipid clusters, and metabolic fingerprints [157]. Recent studies have demonstrated the effectiveness of machine learning algorithms such as Random Forest and Support Vector Machine in identifying unknown protein adulterants in dairy products [158]. The development of explainable artificial intelligence can further screen sparse biomarker combinations, reducing detection costs and improving model generalizability [159]. The second direction is the construction of a systematic validation framework for health functions, which requires combining multi-omics data with in vitro cell models, organoids, and clinical cohort studies to move from correlation to causal inference [160]. Methods such as Mendelian randomization and mediation analysis can be used to validate the actual physiological effects of bioactive peptides, functional oligosaccharides, and other components [161]. Molecular docking simulations can predict the binding affinity of peptides to targets (e.g., angiotensin-converting enzyme), while metabolic pathway enrichment analysis elucidates their mechanisms of action. Ultimately, co-occurrence networks linking dairy components and gut microbiota can be constructed to identify key microbial metabolites (e.g., short-chain fatty acids) that drive host health [28].

**Figure 5:**
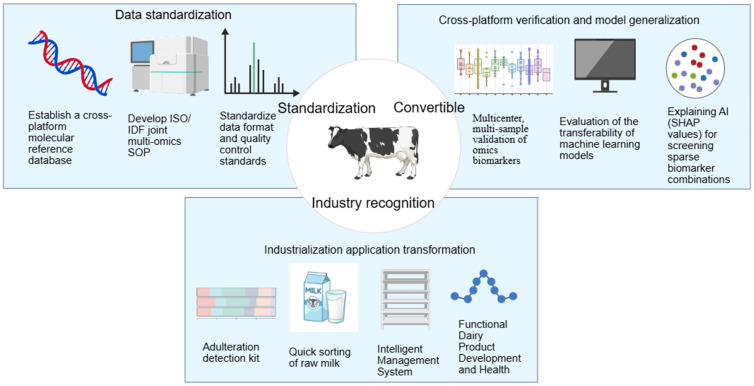
Dairy multi-omics research translation roadmap: standardization, validation, and application. This roadmap illustrates the three sequential stages from research to industrial practice: standardization (data standards and SOPs), validation (biomarker/model robustness assessment with explainable AI), and application (detection kits, rapid sorting, management systems, and functional product development).

Looking to the future, dairy multi-omics research is entering a critical turning point toward industrialization. Priority should be given to the development of real-time detection technologies, transforming validated stable biomarkers into rapid detection kits (e.g., ELISA, lateral flow test strips) or on-line monitoring sensors to meet the demands of production sites and market surveillance [16]. Meanwhile, a comprehensive dairy multi-omics database should be constructed to integrate data including identified bioactive peptide sequences, allergen epitopes, and species-specific peptide biomarkers, which can serve as a unified benchmark for relevant detection. Furthermore, cross-disciplinary cooperation among dairy science, computational biology, clinical medicine, and regulatory policy must be reinforced, and interdisciplinary professionals skilled in both dairy manufacturing and data science should be trained—these two measures are fundamental prerequisites to advance the translation of relevant technologies [28]. Finally, efforts should be made to incorporate multi-omics detection methods into national and international food safety standards (e.g., AOAC, ISO), so that omics markers can become officially recognized markers for adulteration detection and quality assessment [153]. The roadmap at the end of this chapter (Figure 5) summarizes the complete pathway from data standardization and cross-platform validation to industrial application, providing an actionable guide for the implementation of multi-omics technologies in the dairy industry.

### 5.3. Economic and Industrial Feasibility: Barriers to Routine Implementation

Most multi-omics techniques described in this review—particularly high-resolution LC-MS/MS, 4D-label-free proteomics, and metagenomic shotgun sequencing—remain prohibitively expensive and require specialized personnel and infrastructure [162]. This places them beyond the routine quality control budgets of most dairy processing plants, especially small and medium-sized enterprises (SMEs). Furthermore, sample turnaround times (often days to weeks) are incompatible with real-time production line decisions. Therefore, a pragmatic pathway should prioritize (i) converting validated multi-omics biomarkers into low-cost, rapid formats such as ELISA kits, lateral flow strips, or targeted qPCR assays; (ii) developing simplified, portable mass spectrometers or near-infrared sensors calibrated against multi-omics reference data; and (iii) reserving full-scale omics for reference laboratories, dispute resolution, and regulatory enforcement rather than daily screening. Without such translation strategies, the industrial impact of dairy multi-omics will remain marginal.

### 5.4. Multi-Omics Data Fusion and Machine Learning Classification Model Analysis Strategies

Multi-omics integration is not a simple concatenation of data, but rather requires the selection of appropriate fusion levels, algorithmic strategies, and validation schemes based on the research objectives and data characteristics.

#### 5.4.1. Three-Level Architecture for Multi-Omics Data Fusion

Low-level fusion (early integration) directly concatenates raw data or preprocessed features and is suitable for same-platform data (e.g., metabolomics and lipidomics simultaneously acquired by LC-MS); it offers minimal information loss but suffers from the curse of dimensionality, difficulty in aligning heterogeneous data, and a high risk of overfitting [163]. Intermediate-level fusion first performs dimensionality reduction or feature extraction on each omics dataset separately (e.g., PCA, sparse PLS, and autoencoders) before integration, with representative methods including MOFA, DIABLO, and sPLS-DA; this approach is suitable for cross-platform data (e.g., 16S rRNA sequencing + LC-MS metabolomics) and represents the current mainstream strategy for multi-omics integration [164]. High-level fusion (late integration) builds independent models for each omics layer and then combines them via voting or weighted averaging at the decision level, which is suitable for highly heterogeneous data (e.g., genomics + metabolomics + clinical phenotypes); it provides good robustness, such that noise in one omics layer does not easily affect the overall result, but it ignores cross-omics interactions. It is recommended that dairy multi-omics studies prioritize intermediate-level fusion and report the performance improvement (e.g., classification accuracy, AUC) before and after fusion.

#### 5.4.2. Chemometrics and Machine Learning Fusion for Omics Spectral Data

Spectral data generated by techniques such as Raman spectroscopy, near-infrared (NIR) spectroscopy, and hyperspectral imaging (HSI) are essentially “pseudo-omics” data characterized by high dimensionality, high collinearity, and variable signal-to-noise ratios. Commonly used chemometric algorithms include PCA, PLS-DA, SVM, RF, CNN, and one-dimensional convolutional neural networks (1D-CNN). In terms of fusion strategies, inputting spectral features together with mass spectrometry-based omics markers into a model can significantly improve generalization ability. For example, Zhou et al. fused lipid fingerprints and peptide fingerprints from MALDI-TOF MS and combined them with an ensemble learning model, achieving 100% accuracy in organic milk authentication, which was far superior to single-omics models [31].

#### 5.4.3. Role of Explainable Artificial Intelligence (XAI) in Multi-Omics Integration

Although black-box models (e.g., XGBoost, deep learning) offer high predictive accuracy, they cannot meet regulatory requirements for interpretability [163]. Explainable artificial intelligence (XAI) methods, including SHAP, LIME, and attention mechanisms, can be used to identify key markers, simplify detection panels, and improve cross-laboratory reproducibility [165]. In dairy adulteration detection, using SHAP values to select the most stable five peptides allows compression of the model from >200 features to fewer than 10, while maintaining a cross-platform validation AUC > 0.90 [166]. Introducing XAI is a critical step toward moving multi-omics from the laboratory to regulatory acceptance.

#### 5.4.4. Smart Sensing Technologies and On-Line Monitoring: From Laboratory to Industrialization

Smart sensing technologies such as Raman spectroscopy, NIR, HSI, electronic noses, and electronic tongues enable non-destructive, real-time, on-line detection [167]. The integration strategy with multi-omics data involves first discovering markers (e.g., specific metabolites, peptides) through multi-omics, then training spectral models, and finally deploying them on production lines. The AI-integrated Raman platform developed by Bhowmik et al. achieved a classification accuracy exceeding 93% for microbial spoilage in milk, demonstrating commercial potential [168]. However, matrix effects, long-term sensor drift, and insufficient validation in diverse scenarios remain major challenges in real-world production environments. The future path to industrialization should reserve full-scale omics for reference laboratories and dispute arbitration, while converting validated markers into low-cost, rapid detection kits (ELISA, dipsticks, and qPCR) or portable sensors [153,167].

### 5.5. Current Knowledge Gaps and Priority Actions

Despite the significant progress in multi-omics in dairy research, the following four core research gaps, which are directly related to industrial implementation and regulatory acceptance, remain unresolved. These gaps are summarized in Table 5.

## 6. Conclusions

Multi-omics technologies have evolved from single-omics analysis to multi-omics integration, providing systematic molecular insights into the nutritional quality, safety assessment, and health functions of dairy products. In terms of nutritional quality, the combined application of proteomics, metabolomics, and lipidomics has revealed the systematic effects of species, feeding practices, and processing technologies on the molecular composition of dairy products, successfully constructing specific molecular fingerprints for different milk origins.

In terms of safety assessment, adulteration detection methods based on species-specific peptide markers have been able to accurately identify adulteration at extremely low levels. The coupling of metagenomics and metabolomics effectively supports risk warning of spoilage microorganisms and shelf-life prediction.

In terms of health functions, multi-omics technologies have systematically elucidated the targets and signaling pathways of bioactive peptides, clarified the prebiotic pathways of functional oligosaccharides in regulating gut microbiota and producing short-chain fatty acids, and revealed the systemic effects of gut microbiota metabolites as signaling molecules in regulating host immunity and metabolism.

Despite existing research advances, five critical knowledge gaps remain (as detailed in Section 5.5): (1) a lack of publicly available reference datasets with raw LC-MS/MS data from interlaboratory studies; (2) unvalidated false discovery rates for species-specific peptide markers across different geographical regions, heat treatments, and blended dairy products; (3) the absence of randomized controlled trials establishing causal links between multi-omics-identified bioactive peptides and clinical outcomes such as blood pressure reduction or glycemic control; (4) no cost-effectiveness comparison between multi-omics-based adulteration screening and conventional industrial methods (ELISA, qPCR); and (5) a lack of officially recognized regulatory thresholds for machine learning classification algorithms used in dairy authenticity verification.

Future efforts must address economic viability by developing low-cost, rapid assays derived from validated omics biomarkers, making routine application feasible even for small dairies. Future efforts also should prioritize standardized workflows, cross-laboratory validation, and explainable AI-driven biomarker screening, alongside low-cost solutions suitable for small and medium-sized dairies.

## Figures and Tables

**Table 1:** Comparison of multi-omics approaches.

Omics Type	Target Molecules	Application	Key Findings	Advantage	Sample Type/Validation Design
Metabolomics	Organic acids, amino acids	Species discrimination	Identified biomarkers (e.g., uric acid)	High sensitivity	Milk from cow, goat, buffalo; validated by PCA/OPLS-DA cross-validation [17,18,19,20]
Proteomics	Caseins, whey proteins	Allergen profiling	Species-specific peptides	High specificity	Raw/pasteurized milk; validated by LC-MS/MS with spectral library matching [21]
Lipidomics	Fatty acids, phospholipids	Nutritional evaluation	CLA variation	Functional insight	Raw milk from grass- vs. grain-fed cows; validated by GC-MS and orthogonal partial least squares [22,23,24]
Metagenomics	Microbiota	Fermentation impact	Microbial metabolism link	Mechanistic	Fermented milk (dahi, yogurt); validated by 16S rRNA sequencing with repeated sampling [17,20,25]

**Table 2:** Comparison of nutritional characteristics and health effects of different milk sources.

Milk Source Type	Main Nutritional Advantage	Key Functional Components	Potential Health Effects	Sample Type/Validation Designs
Human Milk	Comprehensive essential nutrients, rich in functional proteins	Immune factors, growth factors, hormones, whey proteins	Supports overall infant development, establishes immune system	Pooled mature milk samples (*n* = 30); validated by longitudinal cohort [34,35,36]
Yak Milk	High content of functional proteins, high nutrient density	High levels of lactoferrin, osteopontin, immunoglobulins	Enhances immunity, promotes bone health	Tibetan plateau samples (*n* = 25); cross-validated with ELISA and proteomics [33,37]
Buffalo Milk	High mineral and fat content, high probiotic survival rate	High cholesterol, sphingomyelin, protein, minerals	Supports bone health, beneficial for fermented dairy production	Murrah buffalo milk (*n* = 20); repeated measures across lactation stages [38,39]
Sow Milk	Rich in neurodevelopment-related components and special lipids	N-acetylneuraminic acid, polar lipids, long-chain polyunsaturated fatty acids	Promotes neural development, supports cell membrane function	Colostrum and mature milk (*n* = 15); validated by targeted lipidomics [36]
Cow Milk	High protein content, high yield, wide application	Casein, β-lactoglobulin	Provides high-quality protein	Bulk tank milk from 10 farms; validated by repeated monthly sampling [35]

**Table 3:** Application of multi-omics approaches in dairy safety assessment: authenticity, peptide markers, and allergen risk.

Assessment Area	Omics Technology	Detection Platform	Model/Algorithm	Applicable Products	References
Milk Authenticity and Adulteration Identification	Metabolomics	UPLC-HRMS	PCA, OPLS-DA, SVM	Cow milk, goat milk, horse milk, pasteurized milk, UHT milk	[32,62,63,64,65]
	Proteomics	LC-MS/MS, microLC-IM-QTOF	PCA, PLS-DA	Raw milk, pasteurized milk, milk powder	
	Genomics	qPCR, high-throughput sequencing	Phylogenetic analysis	Raw milk, fermented milk, milk powder	
	Metagenomics	16S rRNA gene sequencing, shotgun metagenomic sequencing	Alpha/beta diversity analysis	Raw milk, cheese, fermented milk	
Species-Specific Peptide Markers	Proteomics/Peptidomics	microLC-IM-QTOF, LC-MS/MS	Database matching, de novo sequencing	Raw milk, pasteurized milk, UHT milk, fermented milk	[66,67,68]
Allergen Detection and Risk Assessment	Proteomics	LC-MS/MS, ELISA	Immunoinformatics algorithms	Raw milk, processed dairy products	
	Metabolomics	UPLC-HRMS	Correlation analysis	Processed dairy products	[69,70,71]
	Metagenomics	16S rRNA gene sequencing, shotgun metagenomic sequencing	Machine learning (e.g., random forest)	Infant formula	

**Table 4:** Multi-omics technologies and strategies for investigating dairy product health functions.

Research Level	Core Omics Technologies/Methods	Research Strategy and Content	Target Health Functions	Sample Type/Validation Design
Molecular Level	Peptidomics, Molecular Docking Simulation	High-throughput mining of milk-derived bioactive peptides; predicting binding modes and structure–activity relationships between peptides and target proteins	Antioxidant, angiotensin-converting enzyme (ACE) inhibition, immunomodulation, etc.	In vitro digests of casein/whey; docking validated with known ACE structure [102,103,104,105,106,107,108,109,110,111,112,113,114,115,116]
Metabolic Pathway Level	Metagenomics, Metabolomics (Integrated Analysis)	Elucidating the effects of functional oligosaccharides on gut microbiota structure and metabolic pathways; revealing the production pathways of beneficial metabolites such as short-chain fatty acids	Prebiotic effects, modulating microbial metabolism to improve gut barrier and immunity, etc.	FOS/GOS intervention in human/infant cohorts; validated by paired metagenomics–metabolomics [117,118,119,120,121,122]
Ecosystem Level	Multi-omics Co-occurrence Network Analysis, Causal Inference Models (e.g., Mendelian Randomization, Structural Equation Modeling)	Constructing interaction networks of dairy components–gut microbes–metabolites–host phenotypes; identifying key microbial taxa and metabolic signaling pathways driving health; moving from association to causal verification	Precision modulation of gut microecology for targeted health interventions	Fermented milk intervention (8 weeks, *n* = 60); validated by Mendelian randomization using LCT variant [123,124,125,126,127,128,129,130]

**Table 5:** The four major unresolved core issues.

Knowledge Gap Category	Specific Problem	Priority Action	References
Data standardization and accessibility	Lack of cross-laboratory, cross-platform, publicly available reference datasets, particularly raw LC-MS/MS data; most studies do not adhere to FAIR principles	Establish a dairy multi-omics reference database, and enforce data sharing and standardized formats	[15,169,170]
Cross-regional validation of markers	The vast majority of peptide markers have only been validated in a single laboratory, in a single breed, or under a single processing condition, and the false discovery rate (FDR) remains unknown	Promote multi-center ring trials, requiring validation by at least three independent laboratories and two or more mass spectrometry platforms	[32,56,171]
Lack of causal evidence	Most studies report associations (e.g., co-occurrence networks, Spearman correlations) and lack of randomized controlled trials (RCTs) to validate the clinical effects of bioactive peptides	Conduct RCTs with multi-omics endpoints, combined with Mendelian randomization for causal inference	[9,172,173]
Regulatory acceptance	There are no officially recognized standards for multi-omics testing, performance thresholds, or guidelines for machine learning model validation	Promote the initiation of projects within international standardization organizations such as AOAC, ISO, and IDF and establish an approval process for multi-omics biomarkers	[172,174,175,176]

## Data Availability

No new data were created or analyzed in this study. Data sharing is not applicable to this article.

## Abbreviations

The following abbreviations are used in this manuscript:

ACE	Angiotensin-Converting Enzyme
CAT	Catalase
DIA	Data-Independent Acquisition
ELISA	Enzyme-Linked Immunosorbent Assay
FOS	Fructo-Oligosaccharides
Foxp3	Forkhead Box P3
GOS	Galacto-Oligosaccharides
GPR	G Protein-Coupled Receptor
GSH-Px	Glutathione Peroxidase
HDAC	Histone Deacetylase
HMOs	Human Milk Oligosaccharides
IgE	Immunoglobulin E
LC-MS/MS	Liquid Chromatography–Tandem Mass Spectrometry
LOD	Limit of Detection
MDA	Malondialdehyde
NMR	Nuclear Magnetic Resonance
NSLAB	Non-Starter Lactic Acid Bacteria
